# Recurrence of Anti-N-Methyl-D-Aspartate Receptor Encephalitis: A Cohort Study in Central China

**DOI:** 10.3389/fneur.2022.832634

**Published:** 2022-03-07

**Authors:** Jilun Feng, Mu Yang, Dingge Cui, Zhi Huang, Tuo Ji, Yajun Lian

**Affiliations:** Department of Neurology, The First Affiliated Hospital of Zhengzhou University, Zhengzhou, China

**Keywords:** anti-N-methyl-D-aspartate receptor encephalitis, anti-NMDA antibody, recurrence rate, relapse, prognosis

## Abstract

**Objective:**

To investigate factors that could impact or predict the probability of anti-N-methyl-D-aspartate receptor (NMDAR) encephalitis recurrence in central China.

**Methods:**

From November 2014 to October 2020, observational data of anti-NMDAR encephalitis inpatients in our institution were collected and analyzed prospectively. The demographics, clinical characteristics, tumor status, lesion locations on MRI and immunotherapies, etc. had entered into a Cox regression model for the identification of the factors associated with relapse-free survival.

**Results:**

We enrolled 113 patients in a row (median age: 28 years, range: 1–61 years). The gender distribution was not statistically significant (*p* = 0.158), with 49 people (43.4%) being female. The median follow-up time was 16 (4–77) months. Among them, 16.8% of patients relapsed. The average interval between recurrences was 8 months (range 3–54 mo). The severity of the initial relapse was less severe than it had been at the start. The first relapse had considerably fewer symptoms (median 2, range 1–6) than the first episode (median 4, range 1–8, *p* = 0.005). The mRS at first relapse (median 3, mean 2.84, range 1–5) had been significantly lower than that at onset (median 4, mean 3.89, range 3–5, *p* = 0.004). The length of hospitalization at first relapse (median 17 days, range 5–46) was significantly shorter than the first episode (median 35 days, range 14–102, *p* = 0.002). In the survival analysis, the risk of recurrence was significantly higher for patients with a brainstem lesion (HR: 4.112, 95% CI: 1.205–14.030; *p* = 0.024) or ≥3 abnormal sites (HR: 2.926, 95% CI: 1.085–7.896; *p* = 0.034) on brain MRI at the first episode. There was no significant difference in neurological outcomes between the recurrent and monophasic groups at the most recent follow-up (mRS 0–2 in 17/19 vs. 86/94; *p* = 0.674).

**Conclusions:**

Anti-NMDAR encephalitis can recur in around one out of every six cases, and symptoms are generally milder than when it first appears. Recurrence is not related to the severity in the acute phase or the prognosis at follow-up. Patients with ≥3 abnormal sites on MRI or lesions located in the brainstem at onset must be alert to the possibility of recurrence.

## Introduction

Anti-NMDA receptor (NMDAR) encephalitis is the most prevalent form of autoimmune encephalitis (AE) and was first reported by Dalmau et al. (1) in 2007. In young adults and children, encephalitis is predominantly found and is mainly manifested as acute or subacute progressive psycho-behavioral abnormalities, seizures, memory deficits, speech disorders/mutism, dyskinesia/involuntary movements, decreased level of consciousness/coma, autonomic dysfunction, and focal central nervous system (CNS) deficit (2, 3). Patients tend to improve after intensive care and immunotherapy, despite the severity of the disease in the acute phase. It had already been reported in the literature that 8–36.4% of patients might relapse after the first episode of anti-NMDAR encephalitis (3–7). Moreover, anti-NMDAR encephalitis could relapse once or multiple times. In contrast to the initial episode, relapses were less severe, more frequently mono-symptomatic, and resulted in fewer admissions to the ICU (3, 8). Early immunotherapy and second-line therapy were proposed to be beneficial in acquiring better results and lower recurrence rates (3, 5). Children who got three or more different immunotherapies at the initial episode had a much lower probability of relapsing, according to an Italian study (9). The clinical features and treatment strategies for anti-NMDAR encephalitis were reported to differ from one country to another (10–12). In 2017, the Chinese Medical Association proposed a domestic consensus on the diagnosis and management of AE, intending to improve the understanding of this disease and determine the prime treatment for Chinese patients (13). We prospectively studied a single-center cohort of patients with anti-NMDAR encephalitis in central China in this research, in order to investigate the factors that may predict and influence the risk of recurrence. The study's findings are likely to be relevant in nations where second-line immunotherapy isn't frequently offered.

## Methods

### Study Design and Population

From November 2014 to October 2020, we prospectively collected inpatients with anti-NMDAR encephalitis admitted to the First Affiliated Hospital of Zhengzhou University. The following were the inclusion criteria (14): (1) acute or subacute onset of one or more of the six major groups of manifestations: psychosis or cognitive deficit, speech disturbances, seizures, movement disorder or involuntary movement, disturbance of consciousness, autonomic dysfunction, or central hypoventilation; (2) CSF tests positive for NMDAR antibodies (cell-based assay); and (3) adequate exclusion of other illnesses. Cell-based assays (CBA) were utilized to detect IgG anti-GluN1 antibodies in the CSF, which used a human embryonic kidney cell line (HEK293) that expresses the receptors as the antigen reactivity substrate (Euroimmun IIFT kits). A tissue-based assay (TBA) used immunohistochemistry on frozen sections of the rat brain (hippocampus and cerebellum) post-fixed with paraformaldehyde as a verified experiment.

The following were the criteria for exclusion: (1) patients with laboratory evidence of infectious encephalitis, for example, viral (toxoplasmosis, rubella, cytomegalovirus, herpes simplex), fungi and Cryptococcus (CSF smear, culture and ink stain), bacteria (CSF smear and culture), parasitic (antibody detection assay), or Mycobacterium tuberculosis (acid-fast stain); (2) patients with a brain tumor or metastasis, alcohol-related encephalopathy, toxic-metabolic encephalopathy, epilepsy or vitamin deficiency, and/or other nervous system disease before the onset of anti-NMDAR encephalitis; (3) other autoimmune encephalitis patients with positive blood and/or CSF laboratory tests: leucine-rich glioma-inactivated protein 1 antibody encephalitis, contactin-associated protein 2 antibody encephalitis, gamma-aminobutyric acid receptors B1/B2 receptor antibody encephalitis, a-amino-3-hydroxy-5-methyl-4-isoxazole-propionic acid receptor antibody encephalitis, voltage-gated potassium channel complex antibody encephalitis, as well as glutamate decarboxylase antibody encephalitis; and (4) patients with <2 months of follow-up or missing key clinical data.

### Data Collection

We prospectively gathered standardized data including (1) epidemiologic data such as sex, age at disease onset; (2) clinical information such as typical symptoms (such as psycho-behavioral abnormalities, memory deficits, speech disturbances, seizures, movement disorders, decreased level of consciousness, autonomic dysfunction, focal CNS deficit), as well as atypical symptoms such as fever, headache, dizziness, and other clinical phenotypes at onset, ICU admission, number of relapses, clinical phenotype at relapse, and time to first relapse. Focal CNS deficit referred to that anti-NMDAR encephalitis can affect the brainstem, cerebellum, etc., causing diplopia, ataxia, and limb paralysis, among other symptoms. (3) Results of ancillary tests such as MRI, EEG and CSF analysis. MRI data were acquired by experienced neuroradiologists utilizing two 3.0 T MRI scanners (Germany Siemens). T1-weighted imaging (T1WI), T2-weighted imaging (T2WI), fluid-attenuated inversion recovery (FLAIR), and diffusion-weighted imaging (DWI) were all common MRI sequences. Gadolinium was used as a contrast agent in contrast-enhanced MRIs. We defined abnormal MRI findings as hyperintensity on T2WI/FLAIR sequences. We counted the number of abnormal sites involved (frontal lobe, parietal lobe, temporal lobe, occipital lobe, insula, hippocampus, basal ganglia, thalamus, corpus callosum, cingulate gyrus, periventricular, cerebellum, brainstem) on brain MRI and split them into two groups (the number of abnormal MRI sites ≥3 and <3). EEG abnormalities had been considered to be slow waves and/or epileptic discharges. In the routine examination of cerebrospinal fluid, leukocytosis was defined as >5 cells/μL, and hyperproteinorrachia had been defined as >45 mg/dL. (4) Immunotherapies which include first-line treatments [corticosteroids, IV immunoglobulin (IVIg), and plasma exchange (PE) alone or in combination], second-line treatments [rituximab (RTX) and cyclophosphamide (CTX) alone or in combination], and long-term immunotherapies [mycophenolate mofetil (MMF) or azathioprine (AZA) >1 year] and other immunotherapy [intrathecal methotrexate (MTX)] (3, 5, 15). The use of three or more distinct immunotherapies was defined as the use of at least three of the above-mentioned medicines (9). At the start of the study, all patients had at least one whole-body tumor screening, which included serological tumor marker screening, chest computed tomography (CT), abdominal, pelvic, and genital area ultrasound. In addition to immunotherapy, patients with tumors received anti-tumor medication. Monophasic (one disease event) or relapsing (≥two disease events, including onset) disease courses were classified. Early treatment was defined as starting immunotherapy within 30 days of onset (16, 17). Recurrence had been defined as new onset or worsening of symptoms occurring after more than 2 months of stabilization or improvement (mRS increased by 1 point or more) (3). During the acute period and at the final follow-up, the modified Rankin Scale (mRS) was used to assess neurological severity. The long-term favorable outcome had been defined as an mRS score ≤ 2, and poor outcome was defined as an mRS score >2 at the last follow-up.

### Statistical Analysis

Because data analysis is dependent on data availability, the denominators in the ‘Results’ can vary. Quantitative variables are expressed in terms of median, mean, and range, while categorical variables are expressed in terms of the number and percentage of subjects in each category. For group comparisons of continuous/ordinal, nominal, and paired data, the Mann-Whitney *U*-test, Fisher's exact test, and Wilcoxon signed-ranks test were utilized. Given the study's major goal, demographics, clinical, and therapeutic data at the time of initiation were used as predictors of recurrence. The follow-up time was defined as follows in the study of survival from the first event: for relapsing patients, the time to first relapse; for relapse-free patients, the time at last follow-up. Candidate predictors of relapse-free survival were first evaluated with univariate Cox-regression analysis and factors significant in the univariate analysis were entered into a multivariable Cox-regression model. The forward LR (forward stepwise regression based on maximum likelihood estimate) method was used to perform multivariable Cox regression analysis. When the number of individuals in the two groups was disproportionate, the validity of survival analysis would be decreased and the bias would be increased. Data were entered into an Excel spreadsheet and analyzed using SPSS IBM 26.0 (RRID: SCR_002865), and figures had been generated using GraphPad Prism 8 (RRID: SCR_002798). *p* < 0.05 was statistically significant.

## Results

Our study comprised 113 people who had anti-NMDAR encephalitis and satisfied the diagnostic criteria proposed by Graus et al. (14). All individuals (100%) were positive for anti-NMDAR antibodies in CSF, and 50% (41/82) were also positive in serum.

The disease first appeared between 2014 and 2020. Forty-nine individuals were female (43.4%), as well as the gender distribution was not statistically significant (Goodness of fit test: *p* = 0.158). The median age at onset was 28 years (mean 29y, range 1–61y, data available in 113/113). There was no difference in age at onset between females (median 24y, mean 27y, range 2y−61y) and males (median 31y, mean 31y, and range 1–61y). (Mann-Whitney *U*-test: *p* = 0.091).

### Clinical Data at First Disease Event

Psychosis (83.2%), cognitive deficit (73%), and seizures (67%) were the most prevalent clinical symptoms of anti-NMDAR encephalitis. Tumors had been detected in 11.5% (13/113) of the individuals, including 6 ovarian teratomas. Two patients developed tumors (nasal myofibroblastoma, lung adenocarcinoma) 3 years after the first symptoms of encephalitis, and one of them relapsed. During the acute phase, approximately 51.3% of patients were admitted to the ICU. Median mRS during the acute phase was 4 (IQR 3–5).

**Figure 1:**
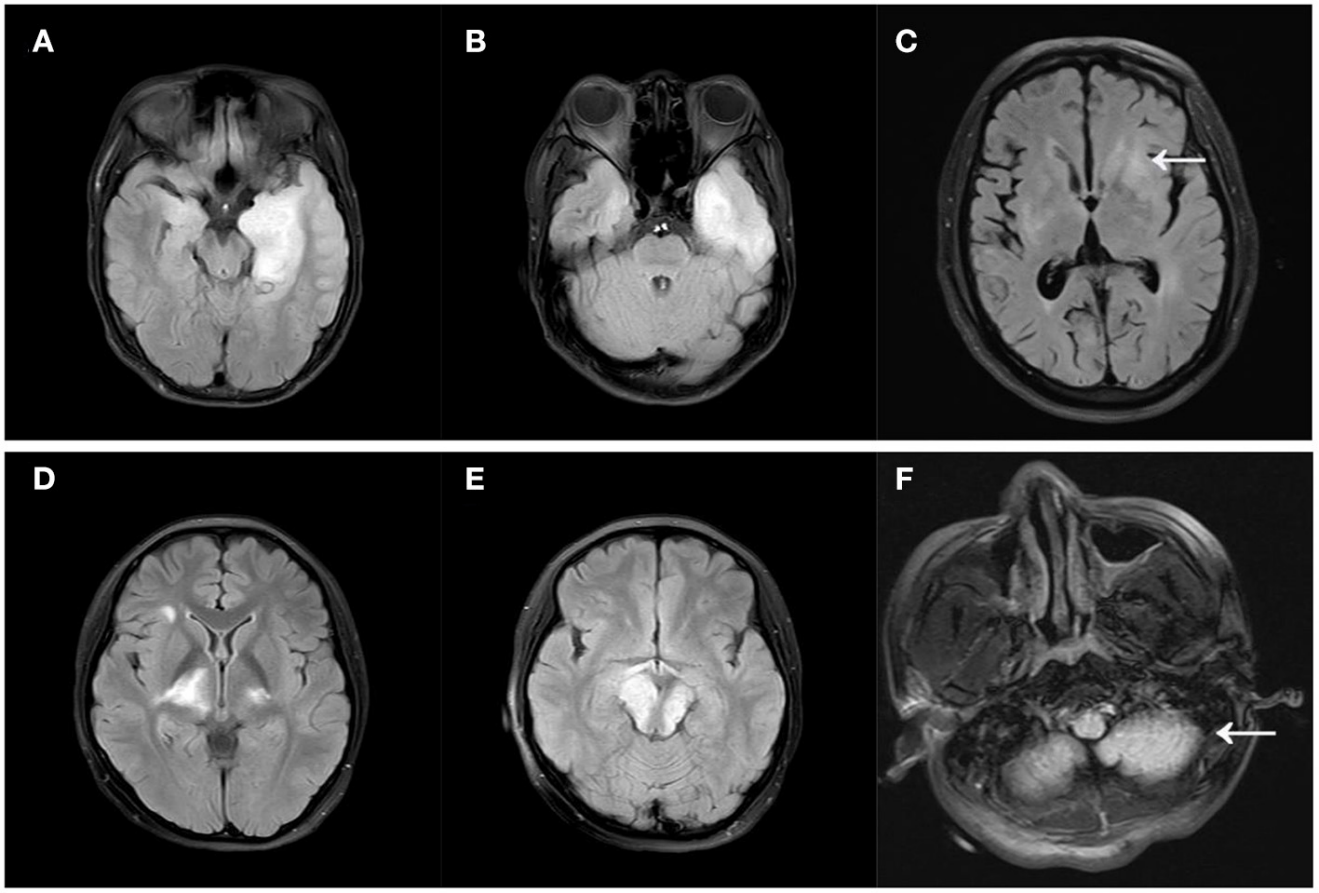
Different MRI types of autoimmune encephalitis. **(A)** Limbic encephalitis; **(B)** cortical/subcortical encephalitis; **(C)** striatal encephalitis (arrow); **(D)** diencephalic encephalitis; **(E)** brainstem encephalitis; **(F)** cerebellar encephalitis (arrow). All MRIs were FLAIR images.

At the time of onset, 111 people underwent a brain MRI, and 60 (54.1%) of them exhibited abnormal T2WI/FLAIR sequence signals, with 27 (24.3%) in the temporal lobe. The frontal, parietal, and occipital lobes, as well as the diencephalon, cerebellum, and brainstem, were all involved (Table 1). An example of brainstem abnormalities is provided in Supplementary Figure 1. As per the anatomical classification of autoimmune encephalitis in the latest guideline (18), the Limbic encephalitis, cortical/subcortical encephalitis, striatal encephalitis, diencephalic encephalitis, brainstem encephalitis, cerebellar encephalitis accounted for 13.5, 33.3, 9.9, 6.3, 6.3, 2.7%, respectively. Figure 1 depicts the different types of MRI scans. Contrast-enhanced MRIs were performed on 53 individuals, with 11 of them showing abnormal contrast enhancement. An example of abnormal contrast-enhanced MRI is provided in Supplementary Figure 2. Abnormal EEG findings were seen in 62.5% of the individuals. Slowing had been the most commonly reported abnormality (45%) and the proportion of epileptiform abnormalities was 25%. Only two people had the delta brush pattern on their EEGs, and one of them is displayed in Supplementary Figure 3. Repeated lumbar punctures were mandatory for diagnosis and analysis, as well as the CSF results at the onset before the immunotherapy had been collected and analyzed. 63.1% of the individuals had leukocytosis (principally lymphocyte and monocytes). The protein level was elevated in 31.2% of the individuals.

**Table 1:** Demographics, data in the acute phase, results of ancillary tests, disease course, as well as outcome in the entire population.

	**Total cohort (*n* = 113)**
**Demographics**	
Year of onset	2014–2016: 22/113 (19.5%);
	2017–2020: 91/113 (80.5%)
Age at onset (years)	Median 28, mean 29.27,
	range 1–61
Proportion of females	49/113 (43.4%)
**Clinical data at first event**	
Clinical symptoms in the acute phase	
Psychosis	94/113 (83.2%)
Cognitive deficit	73/100 (73.0%)
Seizures	75/112 (67.0%)
Prodromal flu-like symptoms	73/113 (64.6%)
Autonomic dysfunction	60/113 (53.1%)
Speech disturbance	55/113 (48.7%)
Disturbance of consciousness	45/113 (39.8%)
Movement disorder	42/113 (37.2%)
Sleep disorder	38/113 (33.6%)
Status epilepticus	35/112 (31.3%)
Focal CNS deficit	31/113 (27.4%)
mRS in the acute phase	Median 4, mean 3.84,
	range 1–5
mRS 3	40/113 (35.4%)
mRS 4	25/113 (22.1%)
mRS 5	40/113 (35.4%)
Associated tumor	13/113 (11.5%)
Admission to the intensive care unit	58/113 (51.3%)
**Auxiliary examination**	
Abnormal brain MRI	60/111 (54.1%)
Frontal lobe	27/111 (24.3%)
Temporal lobe	27/111 (24.3%)
Parietal lobe	19/111 (17.1%)
Periventricular	18/111 (16.2%)
Basal ganglia	11/111 (9.9%)
Hippocampus	9/111 (8.1%)
Occipital lobe	8/111 (7.2%)
Insula	8/111 (7.2%)
Brainstem	7/111 (6.3%)
Thalamus	7/111 (6.3%)
Corpus callosum	6/111 (5.4%)
Cerebellum	3/111 (2.7%)
Cingulate gyrus	2/111 (1.8%)
Abnormal EEG	25/40 (62.5%)
Slowing	18/40 (45%)
Epileptiform discharges	10/40 (25%)
Abnormal CSF	
Leukocytosis	70/111 (63.1%)
Hyperproteinorrachia	34/109 (31.2%)
**Immune therapy at first event**	
Time from onset to first immune therapy	
(days)	Median 20, mean 26.1,
	range 4–194 (d.a. 108/113)
First immune therapy ≤ 30 days from	
onset	82/113 (72.6%)
≥3 different immune therapies	26/113 (23.0%)
First-line immune therapy	108/113 (95.6%)
Corticosteroids	101/113 (89.4%)
IVIG	67/113 (59.3%)
Plasma exchange	20/113 (17.7%)
Second-line immune therapy	5/113 (4.4%)
Rituximab	0/113
Cyclophosphamide	5/113 (4.4%)
Long-term immune modulation	13/113 (11.5%)
Mycophenolate mofetil	7/113 (6.2%)
Azathioprine	6/113 (5.3%)
**Relapses and outcome at last follow-up**	
Length of follow-up (months)	Median 16, mean 22.19
	range 4–77 (d.a.: 113/113)
Proportion of patients who relapsed	19/113 (16.8%)
mRS at last follow-up#	Median 0, mean 0.68,
	range 0–6 (d.a.: 113/113)
mRS 0	75/113 (66.4%)
mRS 1	22/113 (19.5%)
mRS 2	6/113 (5.3%)
mRS 3–6	10/113 (8.8%)

*mRS, modified Rankin Scale; MRI, magnetic resonance imaging; d.a., data available; EEG, electroencephalography; CSF, cerebrospinal fluid; IVIG, intravenous immunoglobulin. Leukocytosis > 5 cells/μL, Hyperproteinorrachia >45 mg/dL. #Total four patients passed away, two of whom died of cancer, one of whom died of a car accident, and the other of unknown causes*.

At the first event, only five people did not receive any immunotherapy. A total of 101 (89.4%) individuals received steroids. IVIG was administered to 67 (59.3%) individuals, and 20 (17.7%) individuals underwent PE. In most cases, it was a combination of repeated usage of steroids and IVIG (61/113, 54%). Second-line immunotherapy was administered in five individuals, which all received CTX, and long-term immunotherapy was used in 13 individuals at the first event. At the first event, 26 (23%) individuals received three or more immunotherapies in total.

Six patients with ovarian teratomas at the time of start had their tumors removed, and one of them had encephalitis recurrence. One patient underwent a resection of meningioma. One patient with gallbladder cancer who underwent cholecystectomy and chemotherapy died of recurrence of gallbladder cancer. One patient had been explored to have lung cancer during hospitalization and died of respiratory failure without anti-tumor therapy because of the poor physical condition. The patient with hepatic hemangioma as well as the patient with ileocecal intraepithelial neoplasia was instructed to undergo regular review. The patient with nasal myofibroblastoma has been receiving regular radiotherapy. One patient had been discovered to have lung adenocarcinoma 2 years after recurrence and was receiving regular chemotherapy.

**Figure 2:**
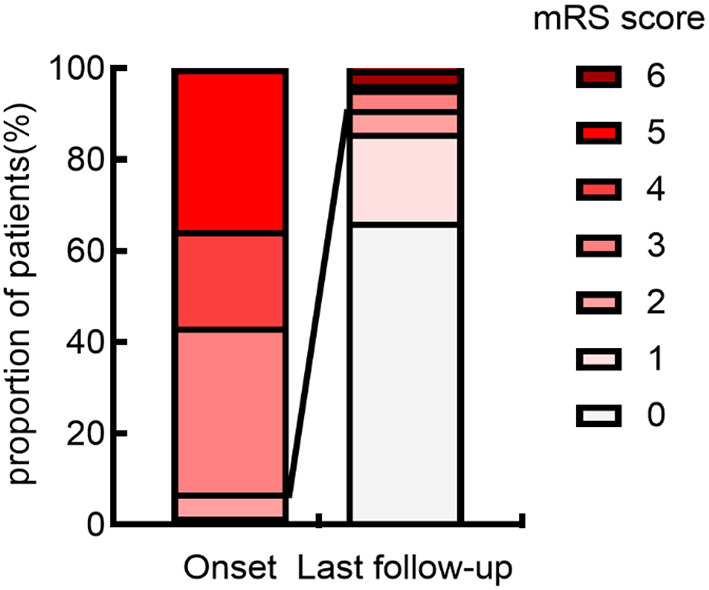
Distribution of mRS scores at onset and last follow-up. The figure demonstrates that the anti-NMDAR encephalitis has a typically good prognosis. The proportion of good neurological outcomes (mRS scores ≤ 2) was significantly higher at last follow-up than at onset. mRS, modified Rankin Scale.

At the last follow-up, 103 (91.2%) individuals had acquired favorable outcomes (median mRS 0, IQR 0–1) (Figure 2). Demographics, clinical data in the acute phase, results of ancillary tests, disease course, and outcome for the entire cohort are presented in Table 1.

### Clinical Data at Relapses

In total, 16.8% (19/113) of the participants relapsed. Seventeen patients experienced one relapse, whereas two had multiple relapses. During the first 12 months, 13 individuals experienced their first relapse. There were 25 years old on average (range 1–52y), and the median time to the first recurrence was 8 months (mean 15 mo, range 3–54 mo; data available in 19/19). Nevertheless, one male patient still relapsed after 4.5 years of onset. Of the relapsed individuals, 9 (47.4%) were female.

In relapsing individuals with available data both at the first and second event, symptom expression at the second event was more limited generally as compared to the initial event (Supplementary Table 1). The number of symptoms at the second event (median 2, range 1–6) was significantly fewer than the first episode (median 4, range 1–8) (Wilcoxon signed-ranks test: *Z* = −2.787, *p* = 0.005). At the second event, the mRS (median 3, mean 2.84, range 1–5) was significantly lower in comparison with the first event (median 4, mean 3.89, range 3–5) (Wilcoxon signed-ranks test: *Z* = −2.869, *p* = 0.004). The first episode lasted 35 (range: 14–102) days in the hospital, while the second incident lasted 17 (range: 5–46) days, which was considerably shorter than the first (Wilcoxon signed-ranks test: *Z* = 3.141, *p* = 0.002). The rate of ICU admission at the second event (6/19) had been lower than that at the first event (10/19), but the difference was not statistically significant (Fisher's exact test: *p* = 0.325).

All 19 relapsed individuals received immunotherapy at the time of their first relapse. Eighteen individuals were re-treated with the first-line immunotherapy, and only two underwent second-line agents (RTX: 1; CTX: 1). Seven individuals acquired long-term MMF treatment and two individuals received long-term AZA treatment.

Clinical data, mRS, hospitalization duration, rate of admission to the intensive care unit as well as immunotherapies at the first and second event in individuals with relapsing anti-NMDAR encephalitis are presented in Supplementary Table 1.

### Survival Analysis of Relapses

**Figure 3:**
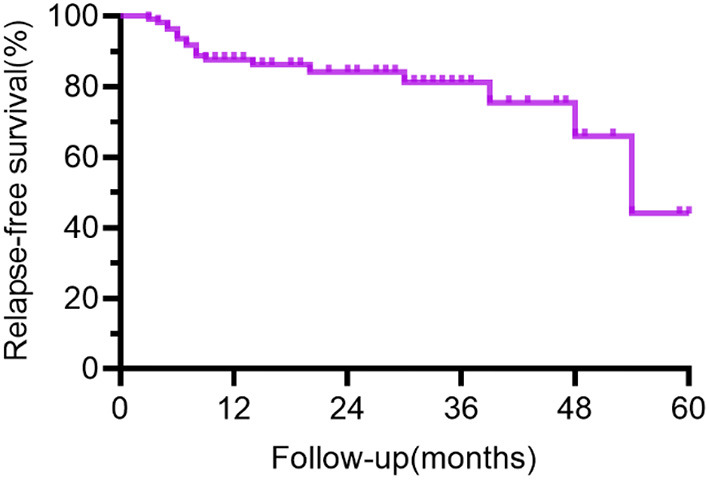
Curve of relapse-free survival for all patients. The time from the first event was plotted on the x-axis, and the % with relapse-free survival was plotted on the y-axis. The figure demonstrates a gradual decrease in recurrence-free survival with longer follow-up in the cohort. The bias toward relapsing disease in the group kept for long-term follow-up explained the low percent of relapse-free survival at 48–60 months in the figure.

In the survival analysis from the first event, the median follow-up time was 14 months (range 3–60 mo). Supplementary Table 2 and Figure 3 demonstrate the clinical features and survival analysis results of individuals with monophasic and relapsing illnesses.

Demographics, clinical symptoms, disease severity (i.e., ICU admission or mRS), tumor status, EEG abnormalities, as well as treatment regimen at the first event were not statistically associated with recurrence.

In the univariate Cox-regression analysis, the risk of relapsing was significantly higher in individuals who manifested as focal CNS deficit (HR: 3.150, 95% CI: 1.278–7.763; *p* = 0.013), when MRI lesions had been located in the frontal lobe (HR: 2.610, 95% CI: 1.059–6.436; *p* = 0.037), brainstem (HR: 6.638, 95%CI: 2.095–21.035; *p* = 0.001), and the number of abnormal MRI sites ≥3 (HR: 3.767, 95% CI: 1.479–9.594; *p* = 0.005) at first disease event.

The risk of recurrence was higher when the lesion was located in the brainstem (HR: 4.112, 95% CI: 1.205–14.030, *P* = 0.024) or the number of abnormal MRI sites was ≥3 (HR: 2.926, 95% CI: 1.085–7.896; *P* = 0.034) when the above factors were included in multivariable Cox-regression analysis (Forward: LR).

**Figure 4:**
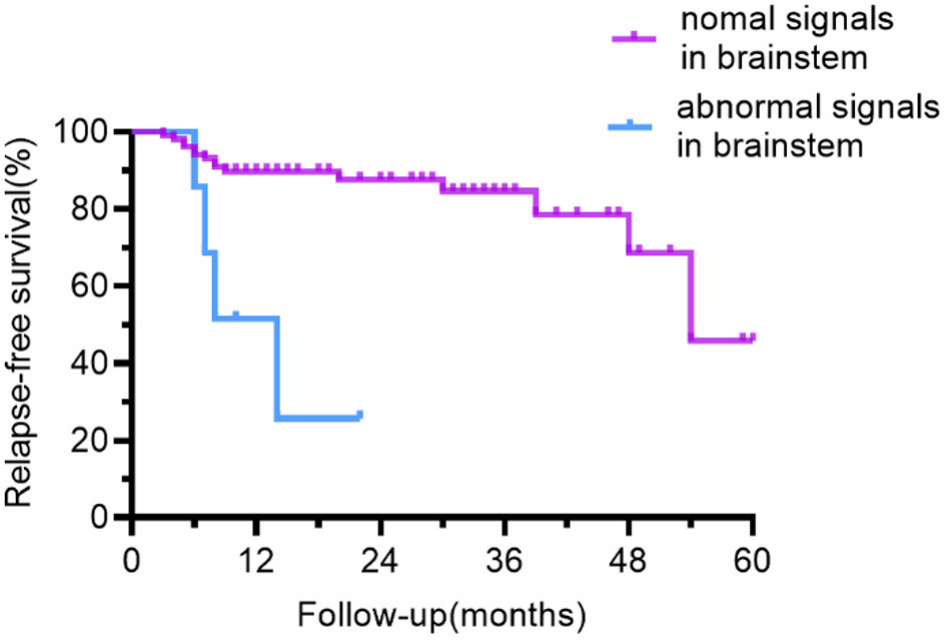
Relapse-free survival curve for patients with abnormal vs. normal brainstem MRI. In contrast to the patients with normal brainstem signals on MRI, patients with abnormal brainstem signals had a lower recurrence-free survival rate.

**Figure 5:**
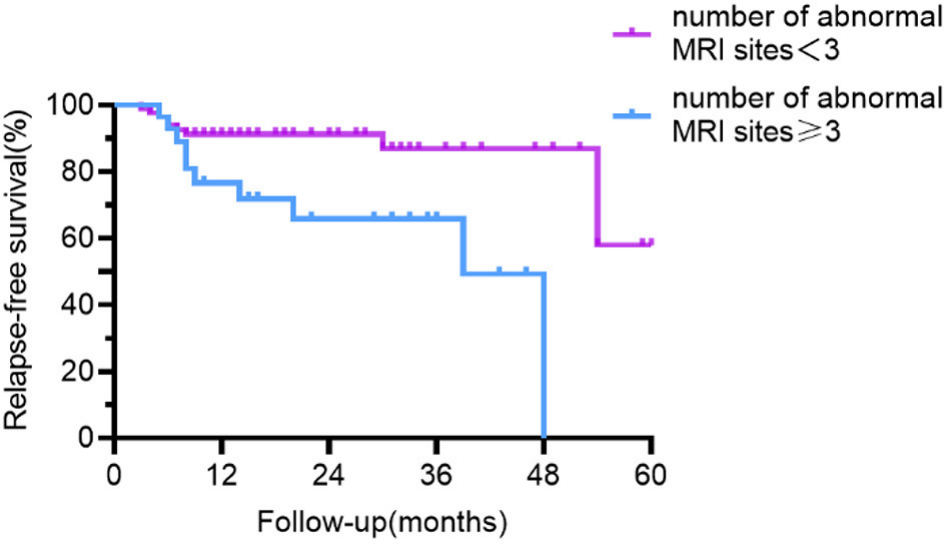
Relapse-free survival curve for patients with abnormal MRI sites <3 vs. ≥3. Compared with patients with <3 abnormal sites on MRI, patients with ≥3 abnormal sites had a lower recurrence-free survival rate.

The outcome of univariate relapse-free survival analysis and multivariable relapse-free survival analysis is presented in Table 2. The relapse-free survival curves of the groups (abnormal vs. normal brainstem MRI, the number of abnormal MRI sites <3 vs. ≥3) are shown in Figures 4, 5, respectively.

**Table 2:** Univariate relapse-free survival analysis and multivariable relapse-free survival analysis.

**Variable**	**Univariate cox-regression**		**Multivariable cox-regression**	
	**HR (95%CI)**	***P***	**HR (95%CI)**	***P***
Focal CNS deficit	3.150 (1.278–7.763)	0.013^*^	2.016 (0.751–5.413)	0.164
Abnormal frontal lobe on MRI	2.610 (1.059–6.436)	0.037^*^	1.077 (0.333–3.482)	0.901
Abnormal brainstem on MRI	6.638 (2.095–21.035)	0.001^*^	4.112 (1.205–14.030)	0.024^*^
Number of abnormal MRI sites ≥3	3.767 (1.479–9.594)	0.005^*^	2.926 (1.085–7.896)	0.034^*^

*HR, hazard ratio; CI, confidence interval*;

* *Indicates P < 0.05*.

### The Outcome in Individuals With Monophasic and Relapsing Disease

Individuals with relapsing and monophasic disease had similar follow-up times (median 29 mo, range 5–77 mo vs. median 15 mo, range 4–60 mo; Mann– Whitney *U*-test: *p* = 0.056). At the last follow-up, there was no significant difference in neurological outcome between relapsing and monophasic patients (mRS 0–2 in 17/19 vs. 86/94; Fisher's exact test: *p* = 0.674).

## Discussion

The clinical aspects of individuals with anti-NMDAR encephalitis were detailed in this study, with an emphasis on factors that may influence and predict the risk of disease recurrence. From a large single-center cohort in central China, the primary findings of the study are as follows: (1) In terms of demographic data, our group differed from Western countries, with more men and a lower tumor prevalence. (2) Approximately one in six patients may experience recurrence and relapses were overall milder than at onset. (3) Patients with ≥ 3 abnormal sites on MRI or lesions located in the brainstem at onset must be alert to the possibility of recurrence.

As per most of the previous works, individuals with anti-NMDAR encephalitis are primarily young females. However, there had been no sex difference in our cohort, which complies with previous Asian countries' reports on adult individuals in Korea (11), on children in Central South China (19) and individuals of all ages with anti-NMDAR encephalitis in Western China (20).

The prevalence of tumors differed between prior studies. According to Titulaer et al. (3), 38% of the individuals had a tumor. Nevertheless, only 11.5% of the individuals in our cohort reported a tumor, and 12.2% of the females had an ovarian teratoma. Other studies of Asian or Chinese individuals have also reported a low frequency of neoplasms [Lim et al. (11), 27.3%; Gong et al. (20), 15.6% Wang et al. (21), 8%; Zhang et al. (22), 8.1%]. The disparity in findings could be attributable to sample sizes and different methods of tumor screening, as well as other factors, which involved epidemiologic reasons and genetic backgrounds, which need to be investigated in the future.

In our study, 16.8% of people relapsed and 1.8% had multiple recurrences, whereas in previous studies, the recurrence rate ranged from 8 to 25%, with one study reaching 36.4% (5–7). The bias toward relapsing disease in the group retained for long-term follow-up explained the low percent of relapse-free survival at 48–60 months in Figures 3–5. As previously described (3, 7, 8), the overall severity of recurrence was milder than the initial time, the frequency of admission to the ICU is lower, and the median length of hospital stay is shortened. It needs to be noted that the recurrence could take a long time interval. Although the majority of patients relapsed within 12 months, one male patient relapsed 4.5 years after the initiation. Individuals with monophasic and relapsing courses did not differ significantly in terms of demographic characteristics or disease severity. Thus, the clinical features and disease severity in the acute phase failed to appear to be credible predictors of recurrence in individuals with anti-NMDAR encephalitis in our cohort, which is consistent with the results derived by a meta-analysis by Nosadini et al. (7).

Anti-NMDAR encephalitis is diffuse encephalitis, according to brain MRI findings. 54.1% of people had MRI abnormalities, the most common of which were frontotemporal, nevertheless, the parietal lobes, occipital lobes, brainstem, cerebellum, and other regions were also involved. The majority of past research has suggested that MRI abnormalities have little bearing on recurrence (8, 9, 20). It is shown only by few studies that individuals with abnormal cranial MRI have a higher frequency of recurrence (23, 24). In our study, MRI abnormalities alone had not been statistically significant (*p* = 0.089), but the risk of relapsing was significantly higher when the number of abnormal sites was ≥3 on MRI. In contrast to those with <3 abnormal sites involved on MRI, the proportion of patients with prodromal symptoms and CSF total protein concentration were higher in those with ≥3 abnormal sites involved on MRI and the differences were statistically significant (Chi-square test, *p* = 0.035; Mann Whitney *U*-test: *p* = 0.019, respectively). We hypothesized that the higher recurrence rate in those with MRI ≥ 3 could be due to a stronger inflammatory response. Similar to other studies of anti-NMDAR encephalitis (2, 6, 15, 25), the brainstem was a rare site. The brainstem abnormalities in our cohort were approximately 6.3% and accounted for 6% in the case series reported by Dalmau et al. (2). Patients having a brainstem lesion on MRI had a greater chance of recurrence. The majority of patients with brainstem lesions experienced dizziness, vomiting, and visual disturbances. Compared with psychiatric symptoms and seizures, the symptoms of this group of patients were less frightening and may cause a delay in diagnosis and treatment. Patients with brainstem abnormalities on MRI took longer to start immunotherapy than those with the normal brainstem but was not statistically significant (Mann Whitney *U*-test: *p* = 0.260). It has also been shown in MOG-positive optic neuritis or myelitis to have a higher recurrence rate when the brainstem is involved (26).

With the increasing awareness of autoimmune encephalitis, the rate of misdiagnosis has gradually decreased (6), and treatment is becoming increasingly timely. According to Gong et al. (20), the proportion of persons treated early in the cohort (between October 2011 and September 2019) was about 58.7%, while the proportion treated early in our cohort increased to 72.6%. Relapse risk was higher in individuals who failed to receive immunotherapy in the first disease event in the work reported by Gabilondo et al. (8). This result failed to be verified in our cohort in consideration of the small number of individuals who didn't receive immunotherapy at onset, possibly due to the recent year of onset. Children who got three or more different immunotherapies at the time of the initial illness event had a much lower probability of relapsing, according to an Italian study (9). In our cohort, both tumor status and treatment regimen were statistically independent of recurrence frequency. This might be because of the low incidence of tumors as well as the diversity of treatment regimens used in the cohort. In our study, those treated with second-line or MMF had similar relapse rates, and only a few people received second-line or long-term non-corticosteroid regimens. This could be due to the fact that RTX and CTX are off-label drugs in China and individuals are concerned about their costs or adverse effects. Meanwhile, a meta-analysis of observational data found that RTX was strongly linked to the non-relapsing disease course (7). Under the circumstances, we recommend more aggressive treatment and advocate the use of RTX in those with brainstem abnormalities or the number of abnormal MRI sites ≥3 if conditions permit.

Anti-NMDAR encephalitis has a typically favorable prognosis, according to prior data (3, 6, 7). At the time of the last follow-up, 66.4% of the individuals had fully recovered, as well as a total of 91.2% of the individuals had a satisfactory neurological outcome. It has been argued in the previous studies that no significant difference in neurological outcome (mRS) between monophasic and relapsing individuals. The utilization of relapse-free survival analysis does effectively control for differences in follow-up duration, concerning the main outcome (relapses). Similar results were achieved in our investigation, implying that a satisfactory conclusion can still be reached in the event of a subsequent disease recurrence.

Our work has a number of limitations. Our cohort may be skewed by more complicated cases because we are a tertiary teaching institution. The number of individuals is limited, and the relapse rate in some patients may be underestimated due to short follow-up periods. When it comes to data analysis, the statistical significance threshold was not corrected for multiple comparisons, and variable selection for multivariable modeling according to univariate significance can be misleading (27, 28). The rough neurological score (mRS) is inappropriate for detecting non-motor symptoms as well as neuropsychological sequelae. Future trials using more specific questionnaires and detailed assessments are needed prospectively (e.g., Neuropsychiatric Inventory, Montreal Cognitive Assessment, Cognitive Impairment Rating Scale, and National Hospital Seizure Severity Scale). Different neuroradiologists assessed the MRI, which could lead to a subjective bias. We only evaluated the EEG reports and there had been little data on EEG. It needs to be pointed out that the findings of the study are most likely only applicable to China or other countries where second-line immunotherapy is not routinely available concerning the very small proportion of second-line therapies applied in our cohort. The majority of relapsed individuals had repeat of first-line treatment rather than second-line treatment, and this is likely to differ in other centers globally where second-line treatment would be used much sooner. Due to the limitation of conditions, we failed to make a validation cohort from another hospital and needed further study. Despite these limitations, our research contributes to our current understanding of anti-NMDAR encephalitis, which is helpful in determining the disease's prognosis.

## Data Availability Statement

The original contributions presented in the study are included in the article/Supplementary Material, further inquiries can be directed to the corresponding author.

## Ethics Statement

This study was approved by the Research Ethics Committee of the First Affiliated Hospital of Zhengzhou University (registration number: 2021-KY-0779-002). The patients/participants provided their written informed consent to participate in this study. For patients under 18 years of age or who were unable to provide consent themselves, written informed consent to participate in this study was provided by the participants' legal guardian/next of kin.

## Author Contributions

JF wrote the main manuscript, analyzed the data, and prepared Tables and Figures. JF, MY, DC, ZH, and TJ took on the task of collecting data and drafting and revising the manuscript. YL designed the study and assigned the work of all authors. All authors reviewed the manuscript. All authors contributed to the article and approved the submitted version.

## Funding

This work was supported by the National Natural Science Foundation of China (No. 81771397).

## Conflict of Interest

The authors declare that the research was conducted in the absence of any commercial or financial relationships that could be construed as a potential conflict of interest.

## Publisher's Note

All claims expressed in this article are solely those of the authors and do not necessarily represent those of their affiliated organizations, or those of the publisher, the editors and the reviewers. Any product that may be evaluated in this article, or claim that may be made by its manufacturer, is not guaranteed or endorsed by the publisher.

## References

[1] Dalmau J Tuzun E Wu HY Masjuan J Rossi JE Voloschin A. Paraneoplastic anti-N-methyl-D-aspartate receptor encephalitis associated with ovarian teratoma. Ann Neurol. (2007) 61:25–36. 10.1002/ana.21050 17262855 PMC2430743

[2] Dalmau J Gleichman AJ Hughes EG Rossi JE Peng X Lai M. Anti-NMDA-receptor encephalitis: case series and analysis of the effects of antibodies. Lancet Neurol. (2008) 7:1091–8. 10.1016/S1474-4422(08)70224-2 18851928 PMC2607118

[3] Titulaer MJ McCracken L Gabilondo I Armangué T Glaser C Iizuka T. Treatment and prognostic factors for long-term outcome in patients with anti-NMDA receptor encephalitis: an observational cohort study. Lancet Neurol. (2013) 12:157–65. 10.1016/S1474-4422(12)70310-1 23290630 PMC3563251

[4] Zekeridou A Karantoni E Viaccoz A Ducray F Gitiaux C Villega F. Treatment and outcome of children and adolescents with N-methyl-D-aspartate receptor encephalitis. J Neurol. (2015) 262:1859–66. 10.1007/s00415-015-7781-9 25987208

[5] Nosadini M Mohammad SS Ramanathan S Brilot F Dale RC. Immune therapy in autoimmune encephalitis: a systematic review. Expert Rev Neurother. (2015) 15:1391–419. 10.1586/14737175.2015.1115720 26559389

[6] Xu X Lu Q Huang Y Fan S Zhou L Yuan J. Anti-NMDAR encephalitis: a single-center, longitudinal study in China. Neurol Neuroimmunol Neuroinflamm. (2020) 7:633. 10.1212/NXI.0000000000000633 31619447 PMC6857906

[7] Nosadini M Eyre M Molteni E Thomas T Irani SR Dalmau J. Use and safety of immunotherapeutic management of N-methyl-d-aspartate receptor antibody encephalitis: a meta-analysis. JAMA Neurol. (2021) 78:1333–44. 10.1001/jamaneurol.2021.3188 34542573 PMC8453367

[8] Gabilondo I Saiz A Galan L Gonzalez V Jadraque R Sabater L. Analysis of relapses in anti-NMDAR encephalitis. Neurology. (2011) 77:996–9. 10.1212/WNL.0b013e31822cfc6b 21865579

[9] Nosadini M Granata T Matricardi S Freri E Ragona F Papetti L. Relapse risk factors in anti-N-methyl-D-aspartate receptor encephalitis. Dev Med Child Neurol. (2019) 61:1101–7. 10.1111/dmcn.14267 31175679

[10] Iizuka T Sakai F Ide T Monzen T Yoshii S Iigaya M. Anti-NMDA receptor encephalitis in Japan: long-term outcome without tumor removal. Neurology. (2008) 70:504–11. 10.1212/01.wnl.0000278388.90370.c3 17898324 PMC2586938

[11] Lim JA Lee ST Jung KH Kim S Shin JW Moon J. Anti-N-methyl-d-aspartate receptor encephalitis in Korea: clinical features, treatment, and outcome. J Clin Neurol. (2014) 10:157–61. 10.3988/jcn.2014.10.2.157 24829602 PMC4017019

[12] Bartolini L Muscal E. Differences in treatment of anti-NMDA receptor encephalitis: results of a worldwide survey. J Neurol. (2017) 264:647–53. 10.1007/s00415-017-8407-1 28154970

[13] NBoCM A. Chinese expert consensus on the diagnosis and management of autoimmune encephalitis. Chin J Neurol. (2017) 50:91–8. 10.3760/cma.j.issn.0253-2727.2017.04.001 28468084 PMC7342722

[14] Graus F Titulaer MJ Balu R Benseler S Bien CG Cellucci T. A clinical approach to diagnosis of autoimmune encephalitis. Lancet Neurol. (2016) 15:391–404. 10.1016/S1474-4422(15)00401-9 26906964 PMC5066574

[15] Dalmau J Lancaster E Martinez-Hernandez E Rosenfeld MR Balice-Gordon R. Clinical experience and laboratory investigations in patients with anti-NMDAR encephalitis. Lancet Neurol. (2011) 10:63–74. 10.1016/S1474-4422(10)70253-2 21163445 PMC3158385

[16] Sartori S Nosadini M Cesaroni E Falsaperla R Capovilla G Beccaria F. Paediatric anti-N-methyl-D-aspartate receptor encephalitis: the first Italian multicenter case series. Eur J Paediatr Neurol. (2015) 19:453–63. 10.1016/j.ejpn.2015.02.006 25792293

[17] Armangue T Titulaer MJ Malaga I Bataller L Gabilondo I Graus F. Pediatric anti-N-methyl-D-aspartate receptor encephalitis-clinical analysis and novel findings in a series of 20 patients. J Pediatr. (2013) 162:850–6.e2. 10.1016/j.jpeds.2012.10.011 23164315 PMC3582718

[18] Abboud H Probasco JC Irani S Ances B Benavides DR Bradshaw M. Autoimmune encephalitis: proposed best practice recommendations for diagnosis and acute management. J Neurol Neurosurg Psychiatry. (2021) 92:757–68. 10.1136/jnnp-2020-325300 33649022 PMC8223680

[19] Wang Y Zhang W Yin J Lu Q Yin F He F. Anti-N-methyl-d-aspartate receptor encephalitis in children of Central South China: clinical features, treatment, influencing factors, and outcomes. J Neuroimmunol. (2017) 312:59–65. 10.1016/j.jneuroim.2017.09.005 28935354

[20] Gong X Chen C Liu X Lin J Li A Guo K. Long-term functional outcomes and relapse of anti-NMDA receptor encephalitis: a cohort study in Western China. Neurol Neuroimmunol Neuroinflamm. (2021) 8:958. 10.1212/NXI.0000000000000958 33589542 PMC8105891

[21] Wang W Li JM Hu FY Wang R Hong Z He L. Anti-NMDA receptor encephalitis: clinical characteristics, predictors of outcome and the knowledge gap in southwest China. Eur J Neurol. (2016) 23:621–9. 10.1111/ene.12911 26563553

[22] Zhang Y Liu G Jiang M Chen W He Y Su Y. Clinical characteristics and prognosis of severe anti-N-methyl-D-aspartate receptor encephalitis patients. Neurocrit Care. (2018) 29:264–72. 10.1007/s12028-018-0536-6 29651625

[23] Zhang JZ Chen Q Zheng P Xie LN Yi XL Ren HT. A comparative analysis of anti-N-methyl-D-aspartate receptor encephalitis with or without abnormal findings on cranial magnetic resonance imaging. Zhongguo Dang Dai Er Ke Za Zhi. (2018) 20:48–51. 10.7499/j.issn.1008-8830.2018.01.010 29335082 PMC7390311

[24] Raja P Shamick B Nitish LK Holla VV Pal PK Mahadevan A. Clinical characteristics, treatment and long-term prognosis in patients with anti-NMDAR encephalitis. Neurol Sci. (2021) 42:4683–96. 10.1007/s10072-021-05174-6 33728548

[25] Wang Y Miao A Shi Y Ge J Wang L Yu C. Influencing electroclinical features and prognostic factors in patients with anti-NMDAR encephalitis: a cohort follow-up study in Chinese patients. Sci Rep. (2020) 10:10753. 10.1038/s41598-020-67485-6 32612192 PMC7329850

[26] Jarius S Kleiter I Ruprecht K Asgari N Pitarokoili K Borisow N. MOG-IgG in NMO and related disorders: a multicenter study of 50 patients. Part 3: Brainstem involvement - frequency, presentation and outcome. J Neuroinflammation. (2016) 13:281. 10.1186/s12974-016-0719-z 27802825 PMC5088671

[27] Sun GW Shook TL Kay GL. Inappropriate use of bivariable analysis to screen risk factors for use in multivariable analysis. J Clin Epidemiol. (1996) 49:907–16. 10.1016/0895-4356(96)00025-X 8699212

[28] Sauerbrei W Perperoglou A Schmid M Abrahamowicz M Becher H Binder H. State of the art in selection of variables and functional forms in multivariable analysis-outstanding issues. Diagn Progn Res. (2020) 4:3. 10.1186/s41512-020-00074-3 32266321 PMC7114804

