# Supplementary material for: Recurrence of Anti-N-Methyl-D-Aspartate Receptor Encephalitis: A Cohort Study in Central China
Source: Front Neurol. 2022 Mar 7;13:832634. doi: 10.3389/fneur.2022.832634 (PMC8959942; doi:10.3389/fneur.2022.832634)
Supplement: Supplementary file 1 [file Image_1.pdf]

Supplementary Figure 1

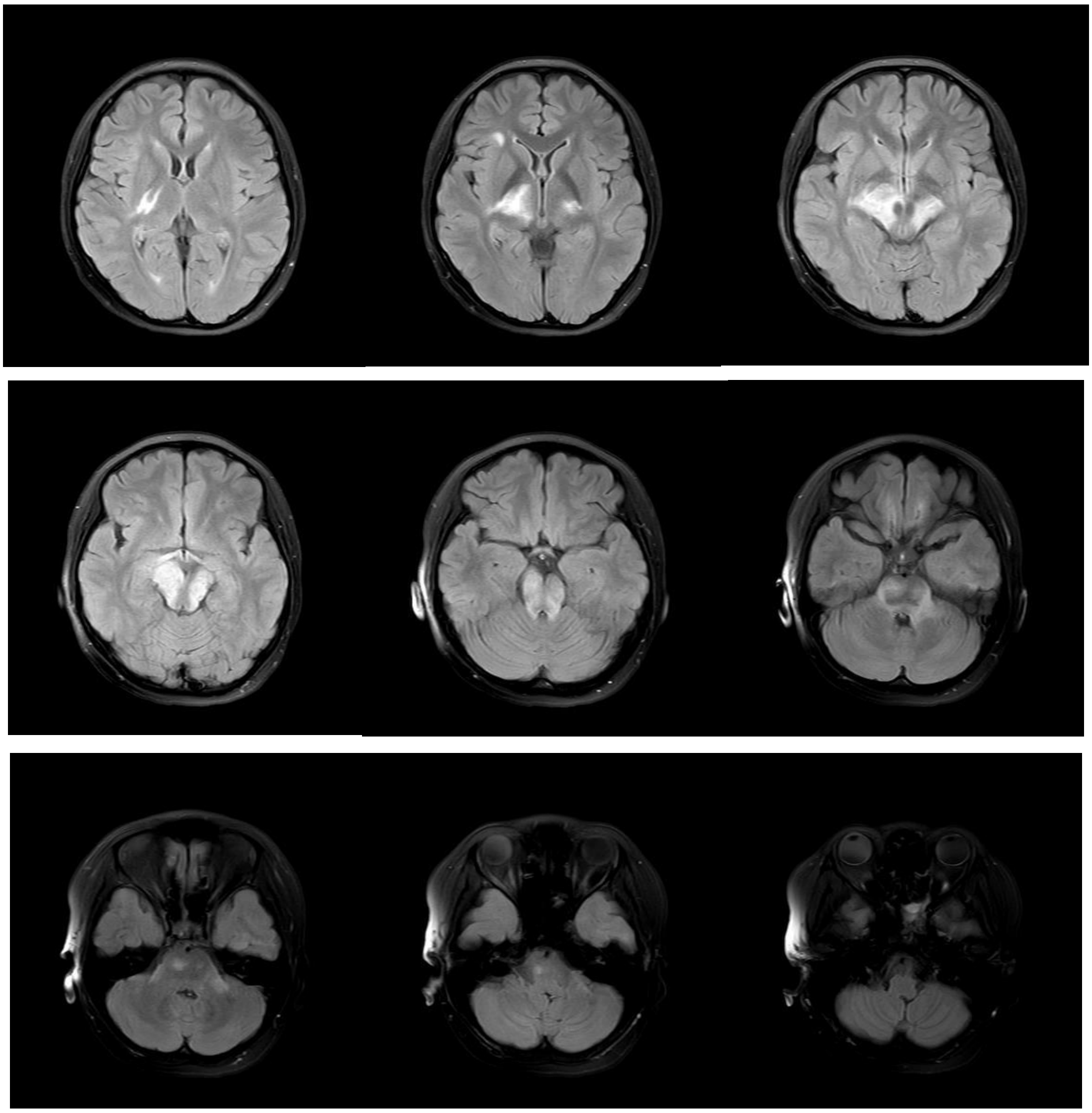

Supplementary Figure 1. Anti-NMDAR encephalitis was detected when a 14-year-old girl was admitted to our hospital with persistent left-sided limb weakness and dizziness. Her brain MRI performed six days after symptom onset showed abnormal signals in basal ganglia, cerebral peduncle, pons, and cerebellopontine junction arm on FLAIR sequence.
