# Supplementary material for: Recurrence of Anti-N-Methyl-D-Aspartate Receptor Encephalitis: A Cohort Study in Central China
Source: Front Neurol. 2022 Mar 7;13:832634. doi: 10.3389/fneur.2022.832634 (PMC8959942; doi:10.3389/fneur.2022.832634)
Supplement: Supplementary file 2 [file Image_2.pdf]

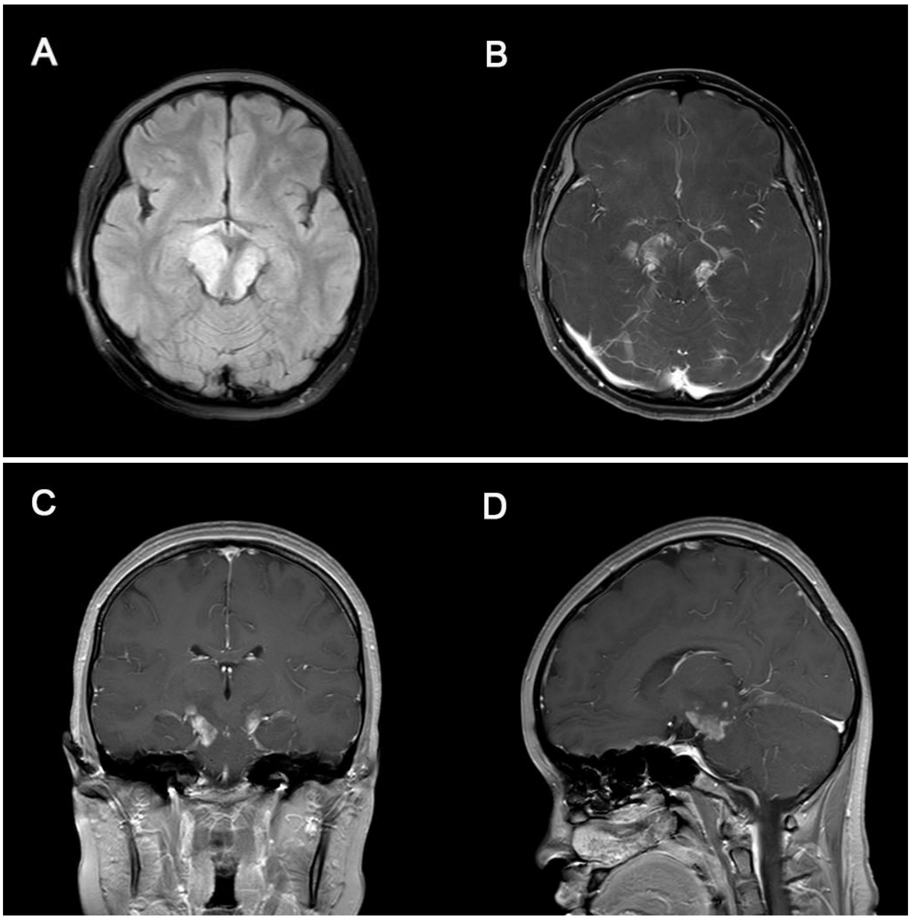

Supplementary Figure 2. The patient had bilateral cerebral peduncles hyperintensities on MRI FLAIR image (A), and abnormal enhancement had been observed in contrast-enhanced MRIs (B horizontal plane, C coronal plane, D sagittal plane).
