# Supplementary material for: Recurrence of Anti-N-Methyl-D-Aspartate Receptor Encephalitis: A Cohort Study in Central China
Source: Front Neurol. 2022 Mar 7;13:832634. doi: 10.3389/fneur.2022.832634 (PMC8959942; doi:10.3389/fneur.2022.832634)
Supplement: Supplementary file 3 [file Image_3.pdf]

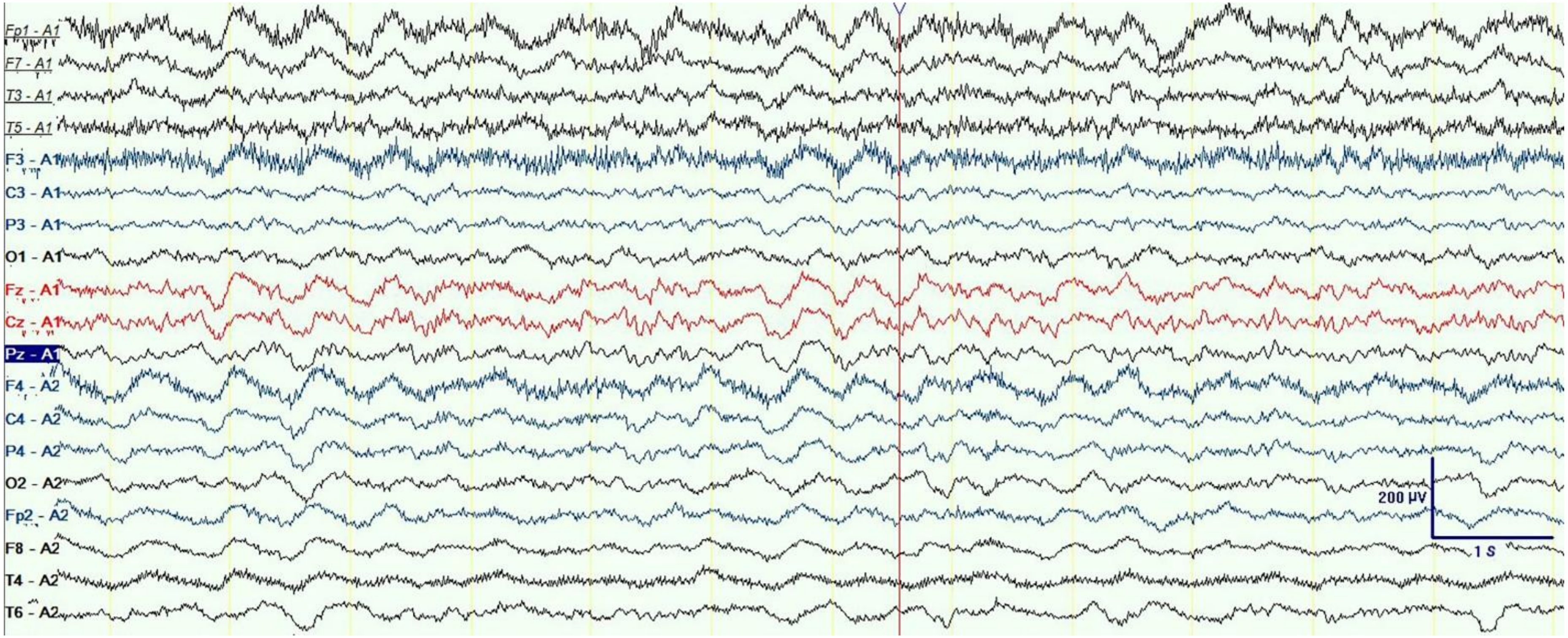

Supplementary Figure 3. Extreme delta brush was seen during the awake phase in a patient with anti-NMDAR encephalitis. The sensitivity was set at 10 μV/mm with low cut and high filter of 0.5 and 70 Hz.
