# Supplementary material for: Recurrence of Anti-N-Methyl-D-Aspartate Receptor Encephalitis: A Cohort Study in Central China
Source: Front Neurol. 2022 Mar 7;13:832634. doi: 10.3389/fneur.2022.832634 (PMC8959942; doi:10.3389/fneur.2022.832634)
Supplement: Supplementary file 4 [file Table_1.DOCX]

**Supplementary Table 1**

|  | **First event (onset)** | **Second event**  **(first relapse)** |
| --- | --- | --- |
| **Clinical symptoms** |  |  |
| Psychosis | 15/19(78.9%) | 9/19(47.4%) |
| Cognitive deficit | 12/19(63.2%) | 12/19(63.2%) |
| Seizures | 11/19(57.9%) | 6/19(31.6%) |
| Prodromal flu-like symptoms | 12/19(63.2%) | 5/19(26.3%) |
| Autonomic dysfunction | 9/19(47.4%) | 3/19(15.8%) |
| Speech disturbance | 6/19(31.6%) | 5/19(26.3%) |
| Disturbance of consciousness | 7/19(36.8%) | 1/19(5.3%) |
| Movement disorder | 8/19(42.1%) | 2/19(10.5%) |
| Sleep disorder | 2/19(10.5%) | 2/19(10.5%) |
| Status epilepticus | 7/19(36.8%) | 2/19(10.5%) |
| Focal CNS deficit | 10/19(52.6%) | 8/19(42.1%) |
| **mRS in the acute phase** | Median 4, mean 3.89, range 3-5 | Median 3, mean 2.84, range1-5 |
| mRS 2 | 0/19(0%) | 6/19(31.6%) |
| mRS 3 | 8/19(42.1%) | 7/19(36.8%) |
| mRS 4 | 5/19(26.3%) | 1/19(5.3%) |
| mRS 5 | 6/19(31.6%) | 3/19(15.8%) |
| **Hospitalization duration** | Median35, mean39.42, range14-102 | Median 17, mean19.58,  range5-46 |
| **Admission to the intensive care unit** | 10/19(52.6%) | 6/19(31.6%) |
| **Immune therapy** |  |  |
| First-line immune therapy | 19/19(100%) | 18/19(94.7%) |
| Corticosteroids | 19/19(100%) | 17/19(89.5%) |
| IVIG | 13/19(68.4%) | 11/19(57.9%) |
| Plasma exchange | 3/19(15.8%) | 1/19(5.3%) |
| Second-line immune therapy | 3/19(15.8%) | 2/19(10.5%) |
| Rituximab | 0/19(0%) | 1/19(5.3%) |
| Cyclophosphamide | 3/19(15.8%) | 1/19(5.3%) |
| Long-term immune modulation | 3/19(15.8%) | 9/19(47.4%) |
| Mycophenolate mofetil | 0/19(0%) | 7/19(36.8%) |
| Azathioprine | 3/19(15.8%) | 2/19(10.5%) |

**Supplementary Table 1.** Clinical data, mRS, hospitalization duration, rate of admission to the intensive care unit as well as immune therapy at first and second event in patients with relapsing anti-NMDAR encephalitis. mRS: modified Rankin Scale; IVIG: intravenous immunoglobulin.
