# Supplementary material for: Recurrence of Anti-N-Methyl-D-Aspartate Receptor Encephalitis: A Cohort Study in Central China
Source: Front Neurol. 2022 Mar 7;13:832634. doi: 10.3389/fneur.2022.832634 (PMC8959942; doi:10.3389/fneur.2022.832634)
Supplement: Supplementary file 5 [file Table_2.docx]

**Supplementary Table 2**

|  | **Monophasic course(n=94)** | **Relapsing course (n=19)** | **p** | **HR (95% CI)** |
| --- | --- | --- | --- | --- |
| **Demographics** |  |  |  |  |
| ≤20y | 24/94(25.5%) | 7/19(36.8%) |  |  |
| 21~39y | 48/94(51.1%) | 8/19(42.1%) | 0.370 | 0.831(0.554-1.246) |
| ≥40y | 22/94(23.4%) | 4/19(21.1%) |  |  |
| **Clinical symptoms in the acute phase** |  |  |  |  |
| Psychosis | 79/94(84.0%) | 15/19(78.9%) | 0.433 | 0.640(0.210-1.953) |
| Cognitive deficit | 61/81(75.3%) | 12/19(63.2%) | 0.599 | 0.778(0.305-1.985) |
| Seizures | 64/93(68.8%) | 11/19(57.9%) | 0.079 | 0.421(0.160-1.105) |
| Prodromal flu-like symptoms | 61/94(64.9%) | 12/19(63.2%) | 0.908 | 0.946 (0.367-2.438) |
| Autonomic dysfunction | 51/94(54.3%) | 9/19(47.4%) | 0.957 | 0.975(0.390-2.436) |
| Speech disturbance | 49/94(52.1%) | 6/19(31.6%) | 0.202 | 0.532(0.202-1.403) |
| Disturbance of consciousness | 38/94(40.4%) | 7/19(36.8%) | 0.927 | 0.957(0.376-2.436) |
| Movement disorder | 34/94(36.2%) | 8/19(42.1%) | 0.535 | 1.343(0.529-3.413) |
| Sleep disorder | 36/94(38.3%) | 2/19(10.5%) | 0.071 | 0.258(0.059-1.120) |
| Status epilepticus | 28/93(30.1%) | 7/19(36.8%) | 0.771 | 1.150(0.448-2.955) |
| Focal CNS deficit | 21/94(22.3%) | 10/19(52.6%) | **0.013*** | 3.150(1.278-7.763) |
| **mRS in the acute phase** | Median 4, mean 3.83, range 1-5 | Median 4, mean 3.89, range 3-5 |  |  |
| mRS≥4 | 54/94(57.5%) | 11/19(57.9%) | 0.657 | 1.233(0.489-3.104) |
| mRS 4 | 20/94(21.3%) | 5/19(26.3%) | 0.635 | 1.282(0.459-3.576) |
| mRS 5 | 34/94(36.2%) | 6/19(31.6%) | 0.959 | 1.026(0.382-2.758) |
| Associated tumor at onset | 10/94(10.6%) | 1/19(5.3%) | 0.612 | 0.593(0.079-4.468) |
| Associated tumor at the last follow-up | 11/94(11.7%) | 2/19(10.5%) | 0.974 | 1.025(0.235-4.469) |
| **Admission to the intensive care unit** | 48/94(51.1%) | 10/19(52.6%) | 0.781 | 1.137(0.460-2.810) |
| **Auxiliary examination** |  |  |  |  |
| MRI(111 available) |  |  |  |  |
| Abnormal MRI | 46/92(50.0%) | 14/19(73.7%) | 0.089 | 2.436(0.873-6.798) |
| Frontal lobe | 18/92(19.6%) | 9/19(47.4%) | **0.037*** | 2.610(1.059-6.436) |
| Temporal lobe | 20/92(21.7%) | 7/19(36.8%) | 0.107 | 2.214(0.842-5.820) |
| Parietal lobe | 14/92(15.2%) | 5/19(26.3%) | 0.221 | 1.904(0.678-5.347) |
| Periventricular | 14/92(15.2%) | 4/19(21.1%) | 0.563 | 1.389(0.457-4.222) |
| Basal ganglia | 9/92(9.8%) | 2/19(10.5%) | 0.656 | 1.398(0.320-6.117) |
| Hippocampus | 9/92(9.8%) | 0/19(0%) | - | - |
| Occipital lobe | 7/92(7.6%) | 1/19(5.3%) | 0.956 | 0.944(0.125-7.130) |
| Insula | 6/92(6.5%) | 2/19(10.5%) | 0.650 | 1.408(0.321-6.173) |
| Brainstem | 3/92(3.3%) | 4/19(21.1%) | **0.001*** | 6.638(2.095-21.035) |
| Thalamus | 4/92(4.3%) | 3/19(15.8%) | 0.217 | 2.203(0.628-7.727) |
| Corpus callosum | 4/92(4.3%) | 2/19(10.5%) | 0.192 | 2.701(0.608-12.000) |
| Cerebellum | 2/92(2.2%) | 1/19(5.3%) | 0.722 | 1.450(0.187-11.219) |
| Cingulate gyrus | 0/92(0%) | 2/19(10.5%) | 0.124 | 3.744(0.696-20.127)- |
| Number of abnormal MRI sites ≥3 | 18/92(19.6%) | 10/19(52.6%) | **0.005*** | 3.767(1.479-9.594) |
| Abnormal EEG | 20/30(66.7%) | 5/10(50%) | 0.625 | 1.283(0.473-3.479) |
| Slowing | 15/30(50%) | 3/10(30%) | 0.337 | 0.513(0.131-2.007) |
| Epileptiform discharges | 6/30(20%) | 4/10(40%) | 0.204 | 2.313(0.635-8.433) |
| Abnormal CSF |  |  |  |  |
| Leukocytosis | 59/93(63.4%) | 11/18(61.1%) | 0.453 | 0.688(0.259-1.826) |
| Hyperproteinorrachia | 32/92(34.8%) | 2/17(11.8%) | 0.110 | 0.299(0.068-1.313) |
| Thyroid dysfunction | 19/77(24.7%) | 3/15(20%) | 0.956 | 1.037(0.282-3.807) |
| Elevated thyroid antibody | 12/46(26.1%) | 2/10(20%) | 0.774 | 0.793(0.164-3.843) |
| **Immune therapy at first event** |  |  |  |  |
| Time from onset to first immune therapy (days) | Median18, mean 24.1, range 4-141 (d.a.89/94) | Median25, mean 35.5, range 10-194 (d.a.19/19) |  |  |
| First immune therapy ≤30 days from onset | 70/94(74.5%) | 12/19(63.2%) | 0.462 | 0.704(0.277-1.792) |
| ≥3 different immune therapies | 19/94(20.2%) | 7/19(36.8%) | 0.166 | 1.936(0.760-4.930) |
| First-line immune therapy | 89/94(94.7%) | 19/19(100%) | - | - |
| Corticosteroids | 82/94(87.2%) | 19/19(100%) | - | - |
| IVIG | 54/94(57.4%) | 13/19(68.4%) | 0.599 | 1.302(0.487-3.478) |
| Plasma exchange | 17/94(18.1%) | 3/19(15.8%) | 0.580 | 1.432(0.401-5.114) |
| Second-line immune therapy | 2/94(2.1%) | 3/19(15.8%) | 0.091 | 3.073(0.836-11.295) |
| Rituximab | 0/90 | 0/19 | - | - |
| Cyclophosphamide | 2/94(2.1%) | 3/19(15.8%) | 0.091 | 3.073(0.836-11.295) |
| Long-term immune modulation | 10/94(10.6%) | 3/19(15.8%) | 0.644 | 1.340(0.387-4.636) |
| Mycophenolate mofetil | 7/94(7.4%) | 0/19 | - | - |
| Azathioprine | 3/94(3.2%) | 3/19(15.8%) | 0.056 | 3.395(0.972-11.864) |

**Supplementary Table 2.** Univariate relapse-free survival analysis. HR, hazard ratio; CI, confidence interval; *Indicates P < 0.05. mRS: modified Rankin Scale; MRI: magnetic resonance imaging; EEG: electroencephalography; CSF: cerebrospinal fluid; d.a.: data available; IVIG: intravenous immunoglobulin. The HRs and CIs are derived from the univariate relapse-free survival analysis. EEG abnormalities were considered to be slow waves and/or epileptic discharges. Leukocytosis>5 cells/uL, Hyperproteinorrachia >45 mg/dL.
